# Identification and verification of a novel epigenetic-related gene signature for predicting the prognosis of hepatocellular carcinoma

**DOI:** 10.3389/fgene.2022.897123

**Published:** 2022-11-30

**Authors:** Chenchen Wang, Chengye Yao, Yan Sun, Jiayi Chen, Yangyang Ge, Yu Wang, Fuquan Wang, Li Wang, Yun Lin, Shanglong Yao

**Affiliations:** ^1^ Department of Anesthesiology, Union Hospital, Tongji Medical College, Huazhong University of Science and Technology, Wuhan, China; ^2^ Institute of Anesthesia and Critical Care Medicine, Union Hospital, Tongji Medical College, Huazhong University of Science and Technology, Wuhan, China; ^3^ Department of Neurology, Union Hospital, Tongji Medical College, Huazhong University of Science and Technology, Wuhan, China

**Keywords:** hepatocellular carcinoma, epigenetic-related genes, prognostic signature, nomogram, immune function

## Abstract

Hepatocellular carcinoma (HCC) is a common malignant tumor with a poor prognosis. Epigenetic dysregulation is now considered to be related to hepatocarcinogenesis. However, it is unclear how epigenetic-related genes (ERGs) contribute to the prognosis of HCC. In this study, we used the TCGA database to identify prognostic ERGs that were differentially expressed in HCC patients. Then, using least absolute shrinkage and selection operator (LASSO) regression analysis, a six-gene signature was constructed, and patients were divided into high- and low-risk groups. Validation was performed on HCC patients from the ICGC database. Patients in the high-risk group had a significantly lower chance of survival than those in the low-risk group (*p* < 0.001 in both databases). The predictive ability of the signature was determined by the receiver operating characteristic (ROC) curve. The risk score was then shown to be an independent prognostic factor for the overall survival (OS) of HCC patients based on the results of univariate and multivariate analyses. We also created a practical nomogram combining the prognostic model with other clinical features. Moreover, functional enrichment analysis revealed that these genes are linked to tumor immunity. In conclusion, our findings showed that a novel six-gene signature related to epigenetics can accurately predict the occurrence and prognosis of HCC.

## Introduction

Hepatocellular carcinoma (HCC) is the most common type of primary liver cancer, accounting for ∼75% of all liver cancers as well as being the fourth leading cause of cancer-related deaths worldwide (Lee et al., 2020). It is caused by chronic HBV or HCV infection, exposure to toxins/environmental factors (alcoholism or aflatoxins), and metabolic disorders [insulin resistance, obesity, type II diabetes, or dyslipidemia in nonalcoholic fatty liver disease (NAFLD)] among others (Liu et al., 2020; Tsilimigras et al., 2020). Only a few patients diagnosed with HCC are in the early stages (BCLC-0 and BCLC-A) and can receive treatment such as local ablation, resection, or orthotopic liver transplantation, according to the BCLC system. However, the treatment of advanced HCC (BCLC-B and BCLC-C) has always been a clinical challenge (Gordan et al., 2020). Furthermore, the high incidence of tumor recurrence (50%–70% 5 years after surgery) impedes improved survival and contributes to poor prognosis (Sun et al., 2021). Due to the significantly increased global burden of HCC and limited treatment options, it is critical to explore new therapeutic targets and develop credible prognostic models.

The advancement of epigenetics has resulted in a significant breakthrough in tumor diagnosis and prediction of clinical outcomes (Deans and Maggert, 2015). Epigenetics refers to changes in cell characteristics that are stable and long-term, rather than to alterations in the DNA sequence (Wu and Morris, 2001; Bird, 2007), such as DNA and RNA methylation, posttranslational modifications (histone and non-histone), and noncoding RNAs (Mohd et al., 2020). During the last decade, several studies have proved that epigenetic signature alterations may lead to a variety of diseases, such as diabetes, cardiovascular diseases, neurological diseases, and cancer (Atlante et al., 2020). A growing body of evidence suggests that abnormal epigenetic factors shape the physiological and pathological processes of HCC and could be used as a type of biomarker for early detection and prognosis of HCC (Grady et al., 2021). Wong et al. (2007) discovered that tissue factor pathway inhibitor-2 (TFPI-2) was frequently silenced by epigenetic alterations in HCC, which include histone deacetylation promoter and methylation. Furthermore, the dysregulation of histone modifications has emerged as an important mechanism for the development of HCC. Previous studies have shown that histone lysine methyltransferase, suppressor of variant 39H1 (SUV39H1), enhancer of zeste homolog 2 (EZH2), euchromatic histone-lysine N-methyltransferase 2 (G9a/EHMT2), and SET domain bifurcated 1 (SETDB1) when significantly upregulated promote the development and metastasis of HCC through epigenetic silencing of key tumor suppressor genes and microRNAs (miRNAs) (Wong et al., 2016; Sun et al., 2017; Wei et al., 2017; Liu et al., 2019).

In this study, the expression of 720 ERGs and their relationship with the prognosis of HCC were systematically examined. We constructed a six-gene prognostic signature and proposed a predictive risk score model to stratify HCC patients. Moreover, we used multiple analysis methods to detect the prognostic value of these genes and constructed a practical nomogram by combining clinicopathological characteristics. Finally, we used the gene set enrichment analysis (GSEA) to investigate the underlying mechanism of our gene signature and the correlation between ERGs and the tumor immune microenvironment.

## Materials and methods

### Data collection

We obtained 424 transcriptome profiling data (normal and tumor specimens) and the corresponding clinical data of 377 HCC patients from the TCGA database as of 1 September 2021 (https://portal.gdc.cancer.gov/), and the same information of another 231 tumor specimens from the ICGC database (https://dcc.icgc.org/projects/LIRI-JP). The information on the clinicopathological characteristics of 377 HCC patients is presented in Supplementary Table S1. We used standardized reading count values. Both TCGA and ICGC data are publicly available. Therefore, permission from the local ethics committee is not required for our study. The present research follows the TCGA and ICGC data access strategies and release guidelines.

### Identification of differentially expressed epigenetic-related genes related to overall survival between tumor and normal samples

We extracted 720 ERGs from the EpiFactors database (https://epifactors.autosome.ru/) and presented them in Supplementary Table S2. The R package “limma” was used to identify differentially expressed genes (DEGs) between HCC samples and normal samples. We used more stringent screening conditions due to a large number of ERGs. The absolute value of the log2 fold change (log2FC) > 2 and false discovery rate (FDR) < 0.05 was adopted as the threshold. To assess the prognostic value of ERGs, we used Cox regression analysis to investigate the relationship between these genes and OS in the TCGA database at *p* < 0.001. We chose the intersection of differential expressed genes and prognostic genes for further investigation. We used the STRING database (https://string-db.org/, version 11.0) to construct an interaction network for the overlapping prognostic DEGs. Then, using a correlation network, we drew the co-expression relationship between these genes.

### Construction and validation of a prognostic epigenetic-related gene signature

Lasso regression, also known as the least absolute value convergence and selection operator, is a method for solving the collinearity of independent variables in linear regression analysis (Tibshirani, 1997). To narrow the range of overlapping genes and establish a prognostic signature, the Lasso Cox regression model (“glmnet” package) was used. Subsequently, a six-gene signature with its corresponding coefficients was constructed, and the penalty parameter (*λ*) was determined using the lowest standard. The “scale” function in R was used to calculate the risk score of each patient: risk score = ∑7iXi×Yi (X: coefficients and Y: gene expression level). Based on the median risk score, the HCC patients from the TCGA database were divided into the high-risk and low-risk groups. Receiver operating characteristic curves were used to predict the accuracy of the prognostic signatures, and Kaplan–Meier survival curves were used to examine the effect of the signature on survival. The OS time of the two subgroups was compared by KM analysis, while a series of R packages including “survminer,” “survival,” and “time-ROC” R packages were used to establish ROC curve analyses for 3 years. The principal component analysis (PCA) and t-distributed stochastic neighbor embedding (t-SNE) were used to determine the principal components. The PCA and t-SNE analyses were performed by the “stats” and “Rtsne” packages based on the gene expression levels in the model. Then, we used the aforementioned formula to obtain the risk score of patients in the ICGC database to verify whether the signature is effective.

### Independent prognostic analysis of risk score

We extracted the clinical characteristics (age, gender, grade, stage, etc.) of patients from both databases. The risk score and clinical variables were then combined to construct a regression model. To identify independent risk factors from the risk score and other clinical characteristics, univariate and multivariate Cox regression models were used at *p* < 0.05.

### Functional enrichment analysis of the differentially expressed genes

We screened out the DEGs between the high-risk and low-risk subgroups using specific criteria (FDR < 0.05 and |log2FC| > 2); the “clusterProfiler,” “ggplot2,” and “enrich plot” packages were executed to perform the GO and KEGG analyses based on the DEGs. The BH method was used to adjust the *p* values. The GO analysis was divided into three parts: biological process (BP), cellular component (CC), and molecular function (MF), whereas the KEGG analysis focused on the metabolic pathways and molecular mechanisms. The ssGSEA in the “GSVA” R package was used to assess the infiltrating level of immune cells and the activity of immune-related pathways [18]. Supplementary Table S3 shows annotated gene set files.

### Building a predictive nomogram in the Cancer Genome Atlas and International Cancer Genome Consortium database

The nomogram transforms the complex regression equation into a visual contour map, making the prediction model more readable, to evaluate the OS probability of individual patients (Park, 2018). In this study, we used Cox regression analysis to select all independent clinicopathological characteristics to build a nomogram that can evaluate the probability of OS in HCC patients at 1, 2, and 3 years.

### External validation of prognostic gene signature

The mRNA expression level (TIMER database, https://cistrome.shinyapps.io/timer/) revealed the abnormal expression of the genes in the prognostic signature. The protein expression profiles (The Human Protein Atlas database, HPA, https://www.proteinatlas.org/) could be used to investigate the expression of proteins represented by genes. Then, we explored genetic alterations in the signature using cBioPortal (http://www.cbioportal.org).

## Results

### Identification of prognostic epigenetic-related differentially expressed genes in the Cancer Genome Atlas database

**FIGURE 1:**
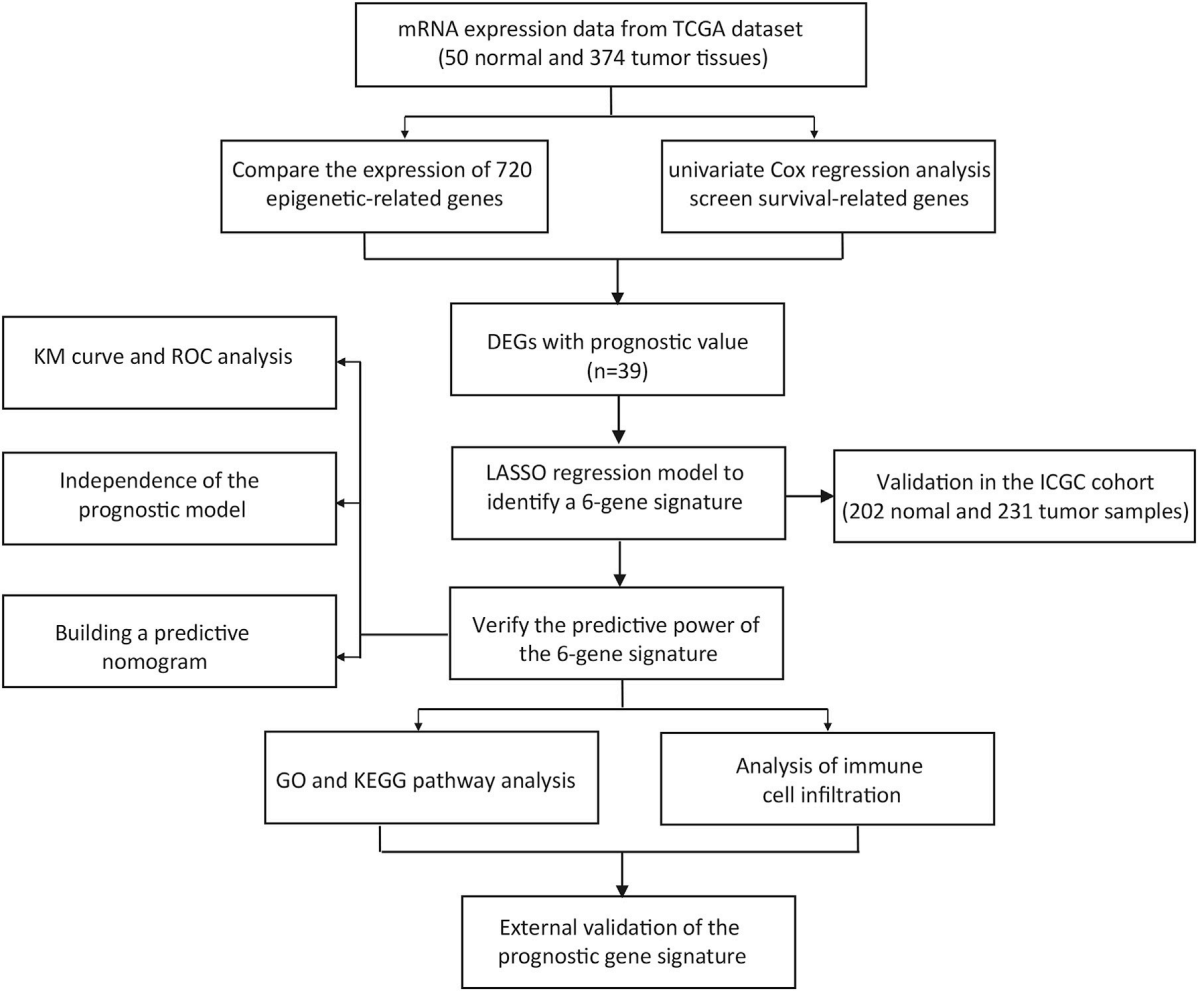
Workflow chart of the study.

**FIGURE 2:**
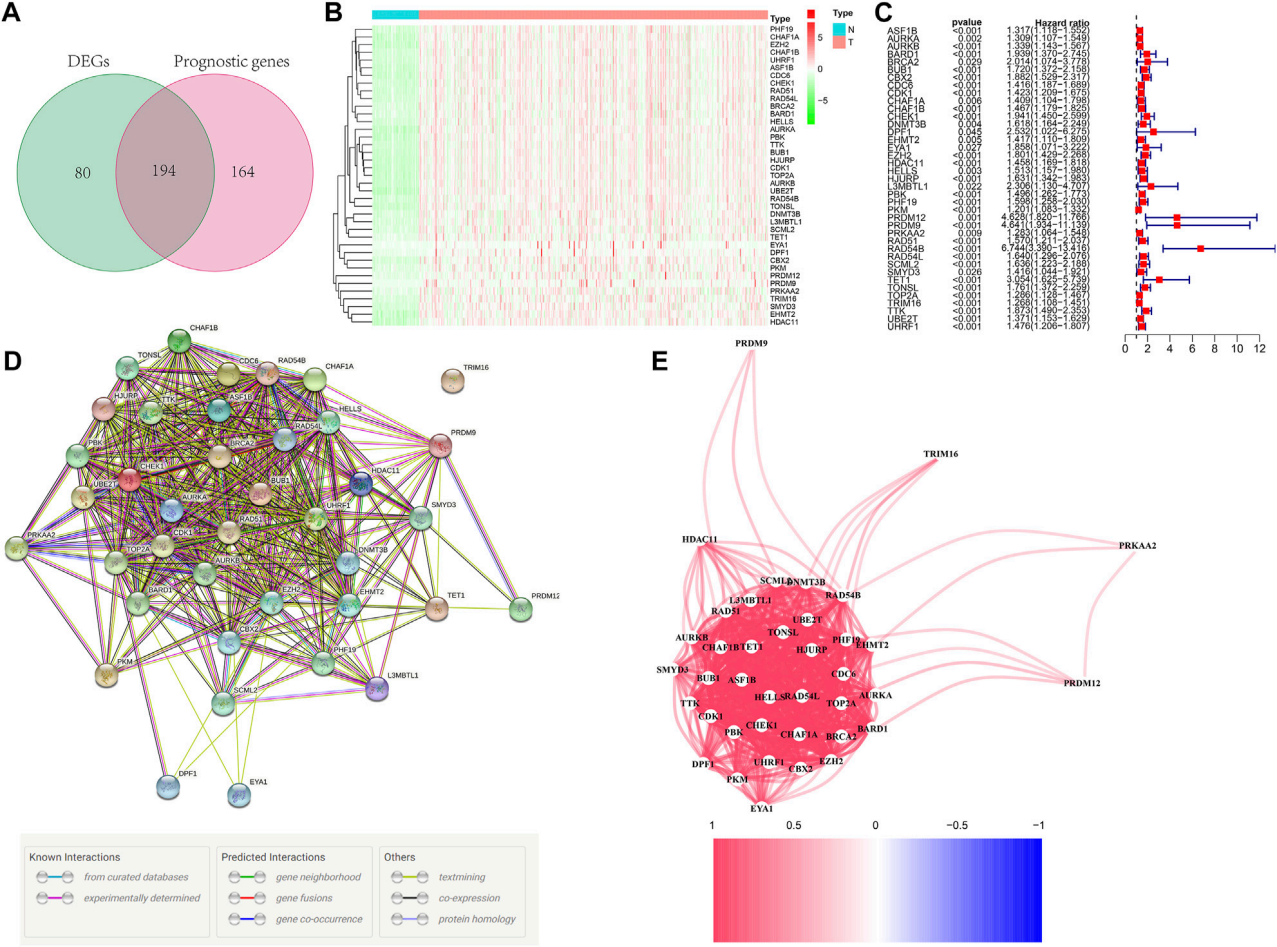
Identification of prognostic epigenetic-related DEGs. **(A)** Venn diagram to identify DEGs related to OS. **(B)** Heat maps of the 39 overlapping genes. **(C)** Results of the univariate Cox regression analysis. **(D)** PPI network indicating interactions among the overlapping genes. **(E)** The correlation network of the overlapping genes.

Figure 1 depicts the workflow chart. We identified 274 DEGs (adjusted *p*-value < 0.05 and |log2FC| > 1) by comparing the expression level of 720 ERGs from 50 normal and 374 tumor samples in the TCGA database. Furthermore, univariate Cox regression analysis was used to screen for prognostic-related genes among them. For further analysis, we identified 194 DEGs that are also related to prognosis (Figure 2A). We chose 39 of these genes for further display due to the large number of prognostic epigenetic-related DEGs. Supplementary Figure S1 shows the complete results. Figures 2B,C show the RNA levels and univariate Cox regression analysis results of these genes. A protein–protein interaction (PPI) analysis was performed to further assess the interactions of these genes, and the results are shown in Figure 2D, as well as the correlation between these genes in Figure 2E. This section may be divided into subheadings. It should provide a concise and precise description of the experimental results, their interpretation, and possible experimental conclusions.

### Development of a prognostic gene model in the Cancer Genome Atlas database

**FIGURE 3:**
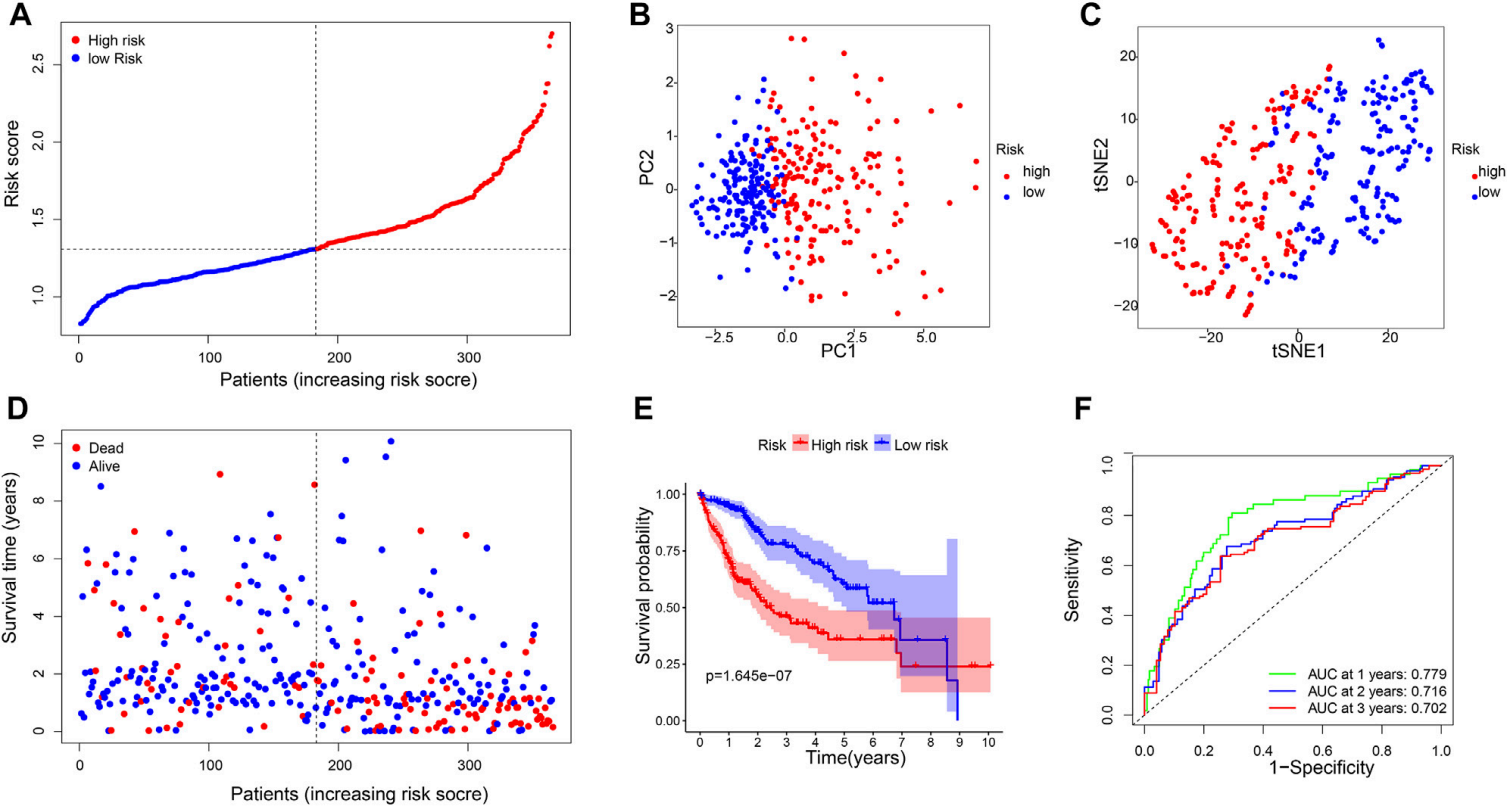
Construction of risk signature genes in the TCGA database. **(A)**. Distribution of patients based on the risk score, **(B)** PCA plot, and **(C)** t-SNE analysis. **(D)** Distributions of survival status (low-risk on the left and high-risk on the right side of the dotted line). **(E)** Kaplan–Meier curves for the OS. **(F)** AUC of time-dependent ROC curves.

A six-gene signature was developed based on the optimal *λ* value using Lasso Cox regression analysis. The risk score was calculated as follows: risk score = (0.22*CBX2 exp.) + (0.18*PPM1G exp.) + (0.28*RAD54B exp.) + (0.07*RUVBL1 exp.) + (0.01*SAP30 exp.) + (0.08*0.08 exp.). Then, 365 patients were divided into the high-risk (*n* = 182) and low-risk (*n* = 183) groups on the basis of the median cut-off expression value (Figure 3A). According to the PCA and t-SNE analyses, the patients in the two risk groups were well separated into different clusters (Figures 3B,C). Compared with the low-risk group, the patients in the high-risk group had a higher probability of death and a shorter lifetime (Figure 3D). Similarly, the KM curve revealed that patients in the high-risk group had significantly shorter OS time (Figure 3E). The ROC analysis was used to estimate the sensitivity and specificity of the gene signature. The area under the ROC curve (AUC) was 0.779 for 1-year, 0.716 for 2-year, and 0.702 for 3-year survival, demonstrating excellent prediction performance of the risk signature (Figure 3F).

### Validation of the risk signature in the International Cancer Genome Consortium database

**FIGURE 4:**
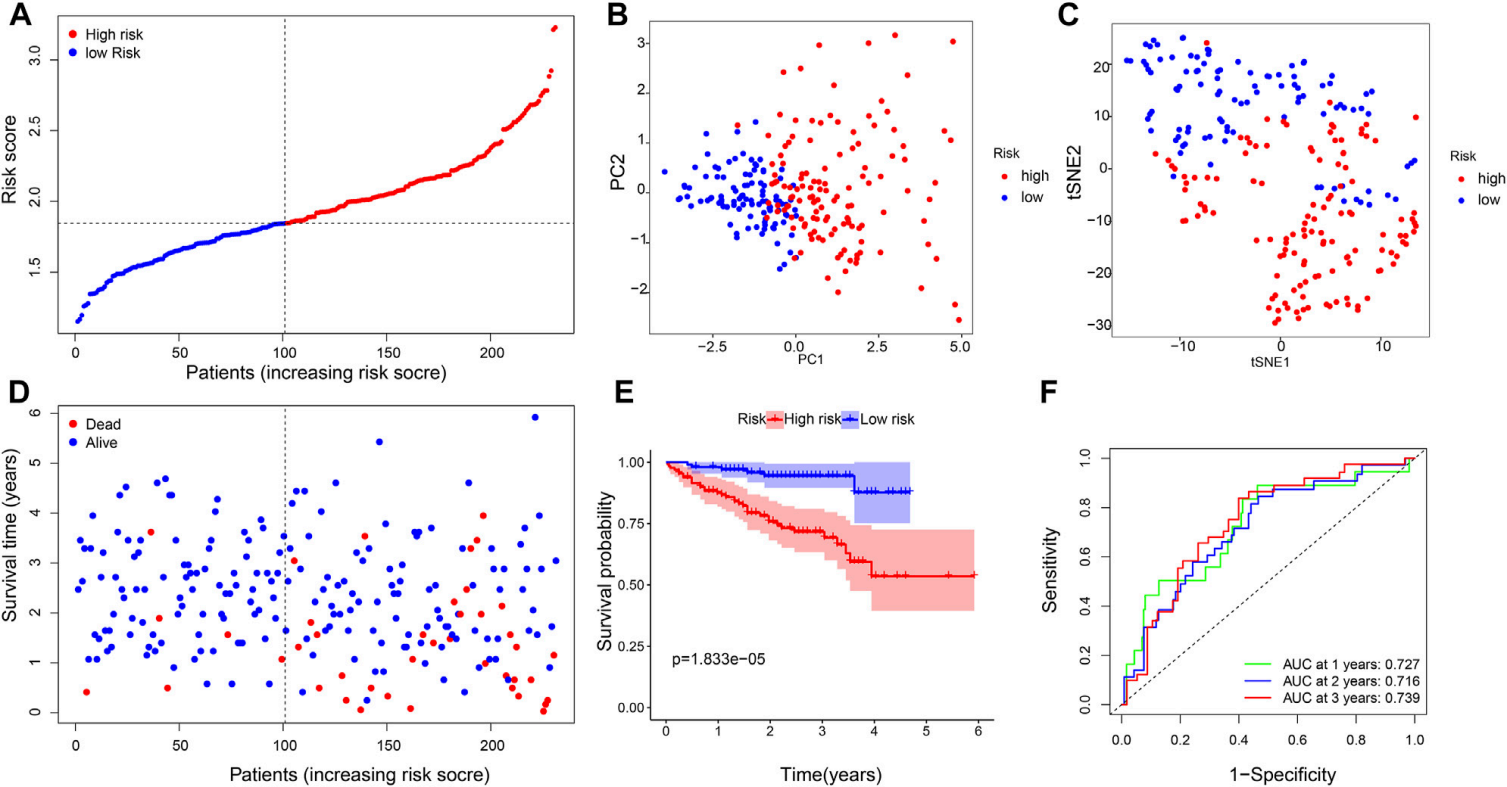
Validation of the six-gene signature. **(A)** Distribution of patients based on the risk score, **(B)** PCA plot, and **(C)** t-SNE analysis. **(D)** Distributions of the survival status (low-risk on the left and high-risk on the right side of the dotted line). **(E)** Kaplan–Meier curves for the OS. **(F)** AUC of time-dependent ROC curves.

We applied the six-gene signature to the ICGC database to test the robustness of our prognostic model. A total of 231 patients from the ICGC database were classified into a high- or low-risk group based on the median value calculated with the same formula as that from the TCGA database (Figure 4A). PCA and t-SNE verified that the patients in the two risk groups were distributed in the discrete direction, which was consistent with the aforementioned results (Figures 4B,C). Moreover, when compared with the high-risk group, patients in the low-risk subgroup had longer survival lifetime and lower mortality than those in the high-risk group (Figure 4D). Furthermore, the KM analysis confirmed that the OS of the high-risk group was significantly lower than that of the low-risk group (*p* = 0.000018, Figure 4E). The AUC on the ROC curve was 0.727 at 1 year, 0.716 at 2 years, and 0.739 at 3 years (Figure 4F), indicating a better predictive efficiency.

### Independent prognostic value of risk signature

**FIGURE 5:**
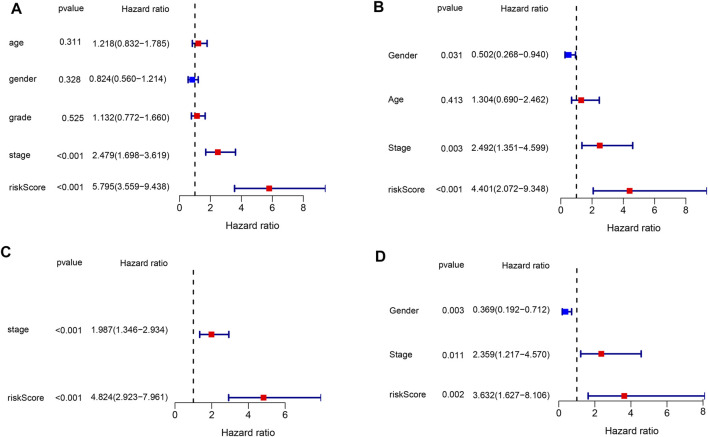
Results of the univariate and multivariate Cox regression analyses in both databases. **(A,B)** Univariate analysis for the TCGA and ICGC databases. **(C,D)** Multivariate analysis for the TCGA and ICGC databases.

The risk score based on the genetic signature was tested using univariate and multivariate Cox regression analyses to assess whether it was an independent prognostic factor among the available variables. In both TCGA and ICGC databases, univariate Cox regression analysis showed that the risk score was significantly associated with poor survival (HR = 5.795, 95% CI: 3.559–9.438 and HR: 4.401,95% CI: 2.072–9.348, Figures 5A,C). The tumor stage was also correlated with OS, while no significant association was found between other clinical features and OS. After adjusting for other confounding factors, the multivariate analysis also proved that the risk score was an independent factor for OS (HR = 4.824, 95% CI: 2.923–7.961 and HR: 3.632, 95% CI: 1.627–8.106, Figures 5B,D) in both cohorts. These results have indicated that the risk score generated from the six-gene signature could serve as an independent factor for the OS of HCC patients.

### Construction of a predictive nomogram in both databases

**FIGURE 6:**
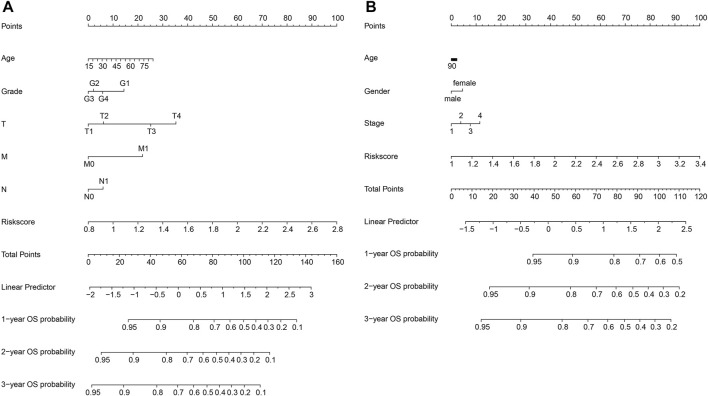
Construction of a nomogram in the TCGA **(A)** and ICGC **(B)** databases.

We constructed a nomogram to predict 1-year, 2-year, and 3-year survival to establish a clinically applicable method to predict the probability of survival of HCC patients. The model incorporates five features of the TCGA database: age, grade, T, M, and N, as well as three features of the ICGC database: age, gender, and stage (Figure 6). Subsequently, a nomogram combining the clinicopathological characteristics with the six-gene signature was developed to enhance the predictive sensitivity and specificity of 1-year, 2-year, and 3-year OS, thereby improving the practicality in the clinical management of HCC patients.

### Functional analyses based on risk signature

**FIGURE 7:**
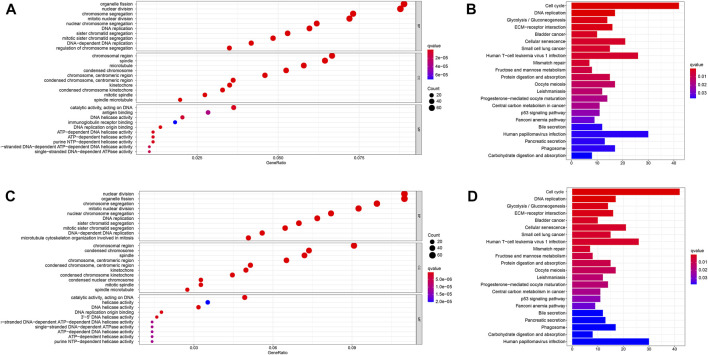
Functional analysis in the TCGA and ICGC databases. **(A)**. Bubble graph for GO enrichment in the TCGA database. **(B)**. Barplot graph for KEGG pathways in the TCGA database. **(C)**. Bubble graph for GO enrichment in the ICGC database. **(D)**. Barplot graph for KEGG pathways in the ICGC database. *q*-value: the adjusted *p-*value.

We then used the “limma” R package to screen out DEGs that differed between the two risk groups to explore the biological functions and pathways related to the risk model (FDR < 0.05 and |log2FC| ≥ 1). Then, GO and KEGG pathway enrichment analyses were performed based on these DEGs. The DEGs were found to be mainly enriched during DNA replication, ATP-dependent DNA helicase activity, and other molecular functions which were associated with epigenetics. Moreover, the DEGs were found to be enriched in the biological processes such as nuclear chromosome segregation and mitotic nuclear division in both databases (*q*: the adjusted *p* < 0.05, Figures 7A,C). Figure 7A reveals that the DEGs were involved in several immune-related molecular functions and biological processes. KEGG pathway enrichment analysis also showed that the cell cycle and human T-cell leukemia virus 1 infection pathway were enriched in both databases (*q* < 0.05, Figures 7B,D).

### Comparison of the immune activity between subgroups

**FIGURE 8:**
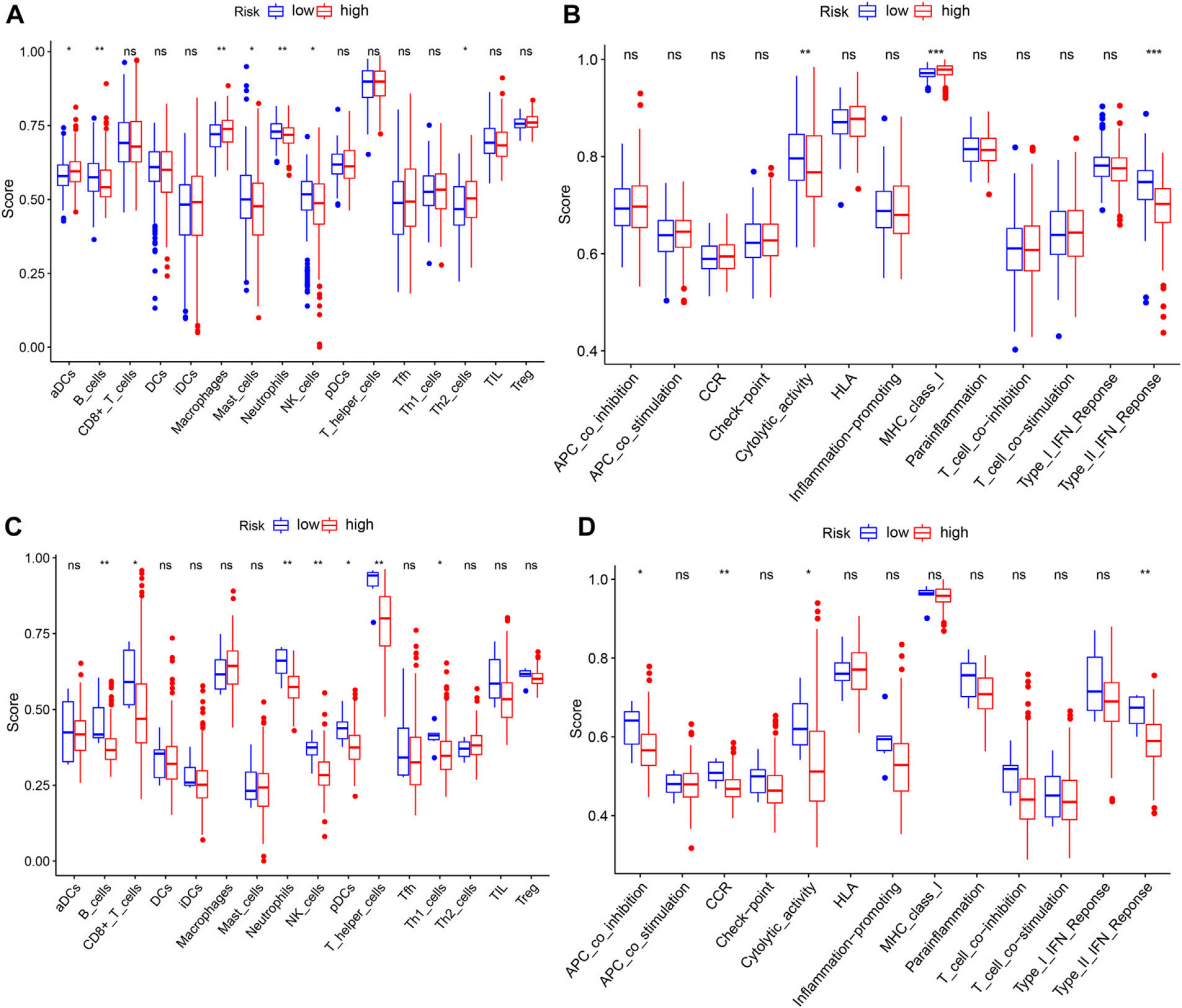
ssGSEA to compare the scores for immune cells and immune-related functions. **(A,C)** Enrichment scores of 16 immune cells in the TCGA and ICGC databases. **(B,D)** Enrichment scores of 13 immune-related functions in the TCGA and ICGC databases. *p*-values shown are ns, not significant; *p < 0.05; **p < 0.01; and ***p < 0.001.

Based on the aforementioned functional analysis, we used ssGSEA to further compare the enrichment degree of different immune cells and immune-related pathways between the two subgroups in the TCGA and ICGC databases. Figure 8A shows that there were significant differences in the level of immune infiltration between the two-risk groups in the TCGA database. The enrichment scores of B cells, mast cells, neutrophils, and NK cells in the high-risk group were lower than those in the low-risk group, whereas the aDCs, macrophages, and Th2 cells showed the opposite pattern. Moreover, in immune function correlation analysis, the scores of MHC class I and type II IFN response and cytolytic activity were significantly different between the two groups (Figure 8B). We confirmed the differences between neutrophils, B cells, cytolytic activity, NK cells, and type II IFN response between the two groups using ssGSEA analysis in the ICGC database (Figures 8C,D). It is worth noting that the enrichment scores of these immune cells and immune function in the high-risk group were downregulated.

### Online database analysis

**FIGURE 9:**
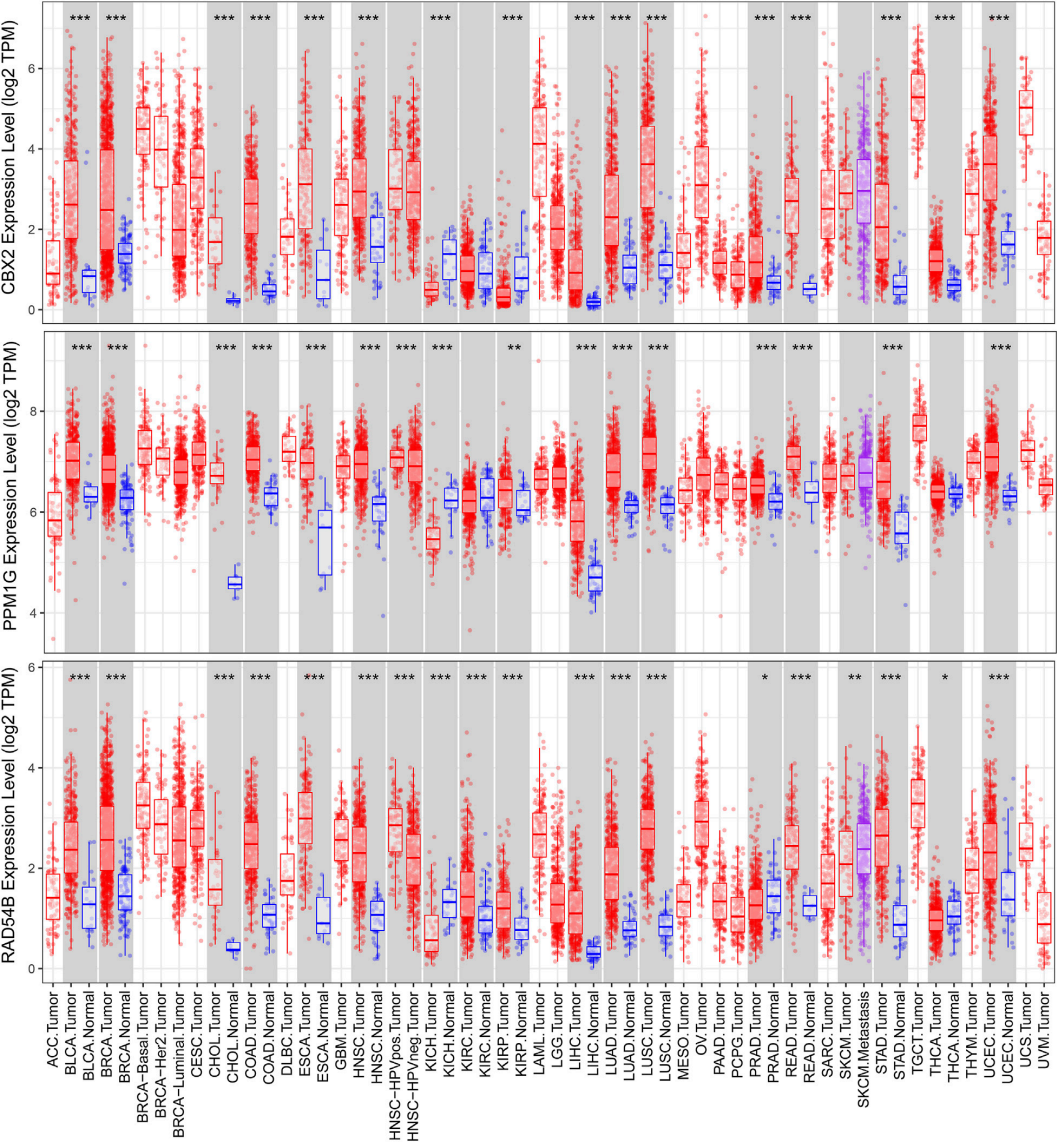
Differential expressions of CBX2, PPM1G, and RAD54B based on the TIMER database.

**FIGURE 10:**
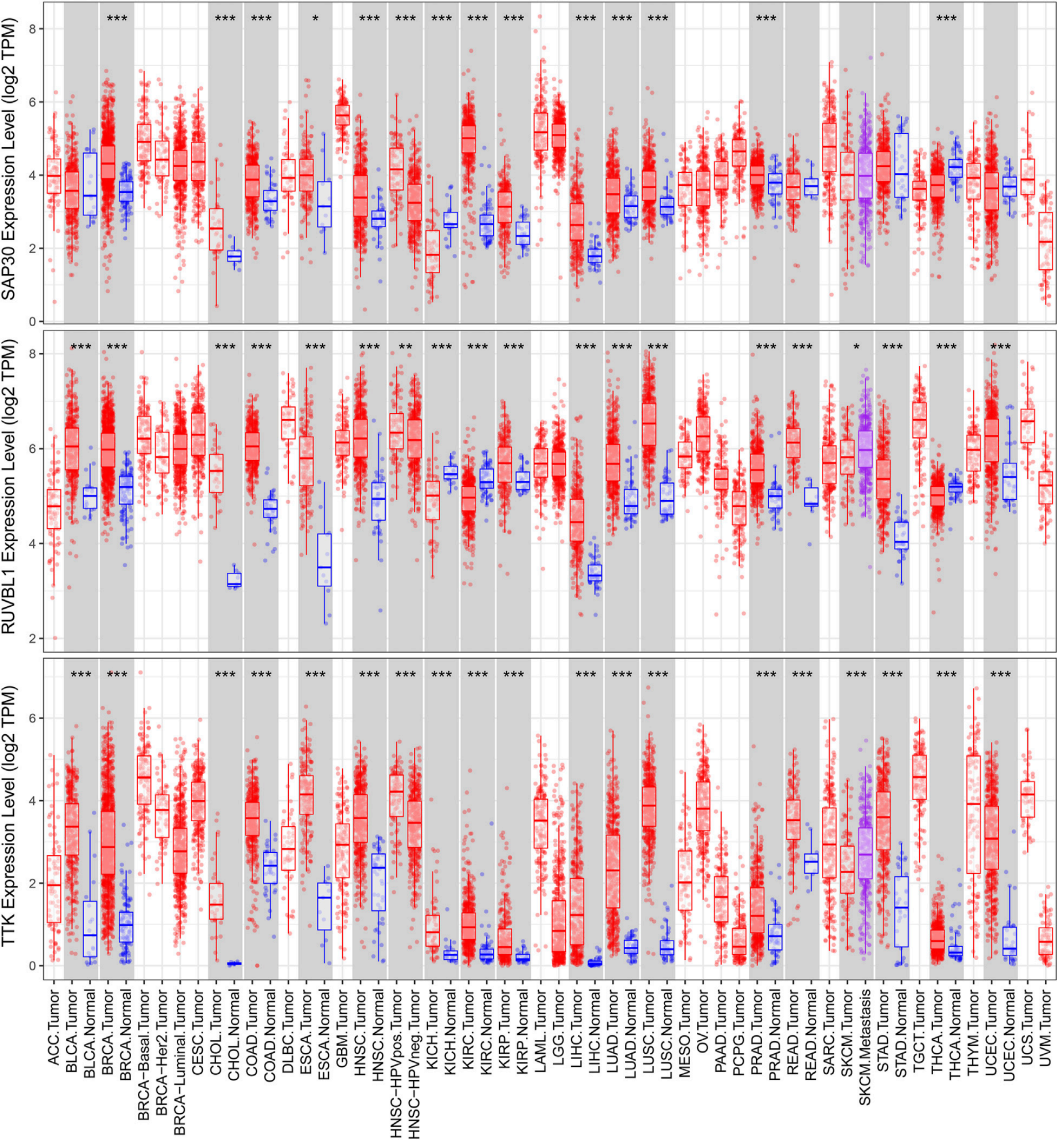
Differential expressions of RUVBL1, SAP30, and TTK based on the TIMER database.

**FIGURE 11:**
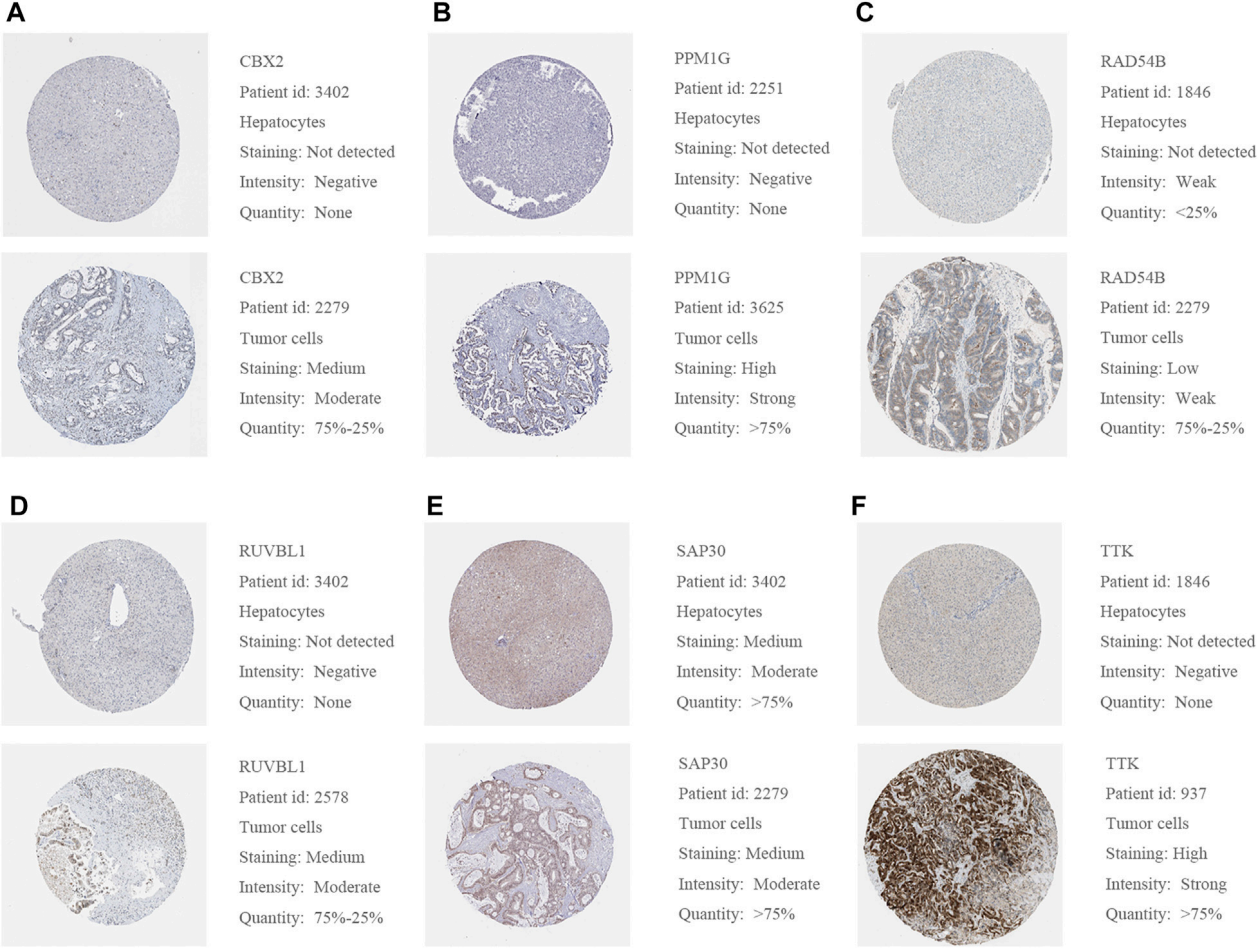
**(A–F)** Representative protein expressions of CBX2, PPM1G, RAD54B, RUVBL1, SAP30, and TTK were explored in the HPA database.

**FIGURE 12:**
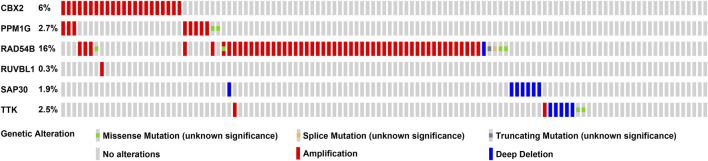
CBX2, PPM1G, RAD54B, RUVBL1, SAP30, and TTK gene expression and mutation analysis.

We used a variety of online databases to explore CBX2, PPM1G, RAD54B, RUVBL1, SAP30, and TTK based on the RNA sequencing data in HCC patients to provide new insights into different prognostic values, expression patterns, and gene mutations of the signature. First, the mRNA expression levels of six genes in various cancer types were analyzed in the TIMER database (Figures 9, 10). The results showed that the expression of these six genes was upregulated in HCC. Furthermore, the expression of these genes was higher in cancer tissues than in normal tissues. Then, we used clinical specimens from HPA to determine the expression of proteins encoded by these six genes for assessing the clinical correlation of these genes. PPM1G and TTK were strongly positive in HCC tissues when compared with their expression level in normal liver tissues, while CBX2, RUVBL1, and SAP30 were moderately positive in HCC tissues (Figures 11A–F). Additionally, we discovered that RAD54B had the most common genetic variations (16%), and the most pronounced change was mutation amplification (Figure 12).

## Discussion

HCC is one of the most life-threatening and heterogeneous malignant tumors in the world (Villanueva, 2019). The poor prognosis and high mortality of HCC are largely due to delayed diagnosis and lack of limited treatment options (Russo et al., 2018; Yang et al., 2020). Finding new prognostic biomarkers to predict outcomes and develop personalized treatment plans for HCC patients is therefore an emergency. Recent studies have demonstrated that the impact of epigenetic-related gene dysregulation on cancer is beyond doubt, but how tumorigenesis is based on the multistep process of epigenome remains to be defined (Nebbioso et al., 2018). Previous research have mainly focused on the function of a single epigenetic-related gene and lacked systematic exploration. In addition, the prognostic value of these genes in HCC is unknown. Thus, we are committed to developing an epigenetic-related gene signature to predict the OS of HCC and providing individualized diagnosis and treatment plans.

We investigated the expression of ERGs in HCC patients and their association with OS in this study. First, we obtained the overlapping genes of differentially expressed and OS-related candidate genes and constructed an interaction network of these candidate genes to narrow the marker range. Then, to assess the prognostic value of these ERGs, we used univariate Cox analysis and LASSO Cox regression analysis to identify a six-gene risk signature in the TCGA database, which we then validated in the ICGC database. The ROC curve further demonstrated the excellent predictive accuracy of the gene signature. The model was then used to divide HCC patients into two risk groups with different survival outcomes in both cohorts. We proved that the six-gene model is an independent prognostic factor that is superior to traditional clinicopathological characteristics for HCC patients. Therefore, we demonstrated that the risk score according to the six-gene signature could be used for the early diagnosis and detection of the prognosis of HCC. Additionally, we constructed a nomogram that is used to provide personalized prediction and treatment strategies for patients. Finally, we conducted a functional enrichment analysis and discovered that the DEGs that differentiated between the high-risk group and the low-risk group were associated with immune-related pathways.

The prognostic signature in this study consisted of six epigenetic-related genes (CBX2, PPM1G, RAD54B, RUVBL1, SAP30, and TTK). The CBX2 gene belongs to the Polycomb group (PcG) of protein family, which is involved in various biological processes, such as cell cycle regulation, cell differentiation, cell senescence, and X chromosome inactivation (Mao et al., 2019; Plys et al., 2019). Previous research has indicated that CBX2 was an epigenetically modified transcription repressor (Chao et al., 2020). Recent studies have linked high CBX2 expression with the poor prognosis of HCC patients. The PPM family is a metal-dependent protein phosphatase. Gene mutations and PPM phosphatase overexpression have been observed in cancer. Hence, PPM phosphatase is now regarded as a promising target for drug therapy (Kamada et al., 2020). The PPM1G is a PPM family member and can dephosphorylate pre-mRNA splicing factors. It is critical in the pathology of many diseases, especially cancer because it influences the diversity of proteins (Liu et al., 2013). Therefore, PPM1G dysfunction may induce cancer progression by affecting pre-mRNA splicing, making it a hot spot in current research (Sun et al., 2016; Lin et al., 2021). The mutation rate of PPM1G was 2.7%, and it overexpressed in HCC patients. RAD54B is a helicase belonging to the SW12/SNF2 superfamily and is involved in DNA homologous recombination and repair (Russo F et al., 2018). Current studies show that abnormal homologous recombination repair is closely associated with human carcinomas (Zhang et al., 2019). Previously, RAD54B was considered a homolog of RAD54 and a cofactor for homologous recombination repair (Yasuhara et al., 2014). In this study, the immunohistochemical results in the HPA database showed that RAD54B is weakly expressed in liver cancer patients, while its gene mutation rate is high (16%). These findings hypothesize that RAD54B plays an unknown role in HCC. At present, small molecule–targeted gene mutants have been developed as a new strategy for cancer treatment (Clarke et al., 2022). The RUVBL1 and RUVBL2 are involved in multiple processes such as transcriptional regulation, chromatin remodeling, telomerase and RNAPII assembly, DNA repair, mitosis, and cell migration and invasion (Yenerall et al., 2020). The expression of RUVBL1 is upregulated in many human tumors such as HCC and is associated with more aggressive cancer types (Lin et al., 2020). The mechanism of RUVBL1 is the primary focus of the current research (Hristova et al., 2020; Lin et al., 2020). The Sin3-associated polypeptide p30 (SAP30) is a component of the human histone deacetylase complex (Zhang et al., 1998). The SAP30 is important for cell growth as well as for affecting gene expression in a promoter-dependent manner. Previous studies have shown that SAP30 has also been conserved in evolution (Huang et al., 2003; Viiri et al., 2009a; Viiri et al., 2009b). There are limited studies on the relationship between SAP30 and liver cancer. However, our results on immunohistochemistry show that SAP30 is moderately expressed in HCC patients, which warrants further studies. The TTK (Mps1) is found in the spindle, which participates in mitotic spindle organization and biogenesis (Xie et al., 2017). Except for the testis and placenta, TTK is almost undetectable in normal organs. However, TTK has been detected in various malignant tumors, such as glioblastoma, thyroid cancer, breast cancer, and other cancers (King et al., 2018; Zhang Z et al., 2019). The effect of TTK in the progression of breast cancer has been shown, while its role in liver cancer has received little attention.

The objective of the epigenetics study is actually to investigate how nongenetic factors act on the genome to influence gene expression and phenotype (Hardy and Mann, 2016; Lu et al., 2020). Therefore, epigenetics can help to explore the mechanisms behind disease phenotypes and may provide new clues to the basis of interpatient variability in disease progression. We sought to investigate the molecular basis of cell homeostasis loss by elucidating the epigenetic modifications associated with liver cancer. However, it is critical to note that our understanding of cancer epigenome alterations is in its very early stages. Our understanding of how epigenetic disorders trigger tumorigenesis is dwarfed by the body of knowledge that we have built on changes in the cancer genome. Based on the DEGs between different subgroups, we conducted GO and KEGG, then we unexpectedly found that some immune-related functions and pathways were enriched. Thus, we hypothesized that epigenetics may be closely related to tumor immunity. We found low levels of antitumor infiltrating immune cells, such as B cells, CD8^+^ T cells, neutrophils, and NK cells, indicating that the immune function of the high-risk group in the TCGA database is generally compromised. This has also been supported by the ICGC database. The type II IFN response pathway and cytolytic-activity pathway were also inhibited in the high-risk group, according to the immune function analysis. According to the study findings, the poor prognosis of HCC patients with high risks may be related to the low level of antitumor immunity. Single-cell sequencing technology can investigate the different biological properties of single cells in complex tissues, and it has been extensively widely used in various diseases, especially tumors (Song et al., 2022). Single-cell multi-omics technologies are emerging to detect single-cell genomes, single-cell transcriptomes, single-cell epigenomes, and single-cell proteomes to help better understand the cell-to-cell differences (Song et al., 2022). Moreover, the comprehensive mapping of the immune cells by single-cell sequencing enriches the understanding of HCC immunity (Li et al., 2022). We can further investigate the relationship between our gene signature and immunity through single-cell multi-omics technologies. The current understanding of cancer epigenome changes is still in its early stage, particularly the mechanism of epigenetics in the occurrence and development of liver cancer is not clear. Our study screened out six genes from many epigenetics-related genes and evaluated their prognostic value to provide theoretical support for follow-up studies. Nevertheless, there are several limitations to this study. First, our prognostic models are identified and validated on the basis of the retrospective data from public databases. There is a need for more expected real data to demonstrate their clinical utility. Second, the relationship between the risk scores and immunization activities has not been established.

In conclusion, our study showed that epigenetics is closely related to hepatocellular carcinoma because many epigenetic-related genes were differently expressed between normal and HCC tissues. Furthermore, in the TCGA and ICGC databases, the risk score generated from our six epigenetic-related genes signature was an independent risk factor for predicting prognosis. Functional analysis of different risk groups revealed an association with tumor immunity. Our findings establish a new genetic signature for predicting the prognosis of HCC patients and providing an important foundation for future studies on the underlying mechanism between epigenetic-related genes and tumor immunity.

## Conclusion

We identified a valid, innovative, and reliable six epigenetic-related gene prognostic models (CBX2, PPM1G, RAD54B, RUVBL1, SAP30, and TTK) to predict HCC patient outcomes. Our signature was an independent and important risk factor for HCC. Moreover, a nomogram combining our prognostic model and clinicopathological features was constructed, which could increase the usefulness in the clinical management of patients. Furthermore, the correlation between epigenetics and tumor immunity found in functional analysis can be used as a clue for in-depth research.

## Data Availability

The raw data supporting the conclusion of this article will be made available by the authors, without undue reservation.

## Supplementary material

SUPPLEMENTARY FIGURE S1 Identification of the prognostic epigenetic-related DEGs.
