# Supplementary figures and images for: Identification and verification of a novel epigenetic-related gene signature for predicting the prognosis of hepatocellular carcinoma
Source: Front Genet. 2022 Nov 30;13:897123. doi: 10.3389/fgene.2022.897123 (PMC9748485; doi:10.3389/fgene.2022.897123)

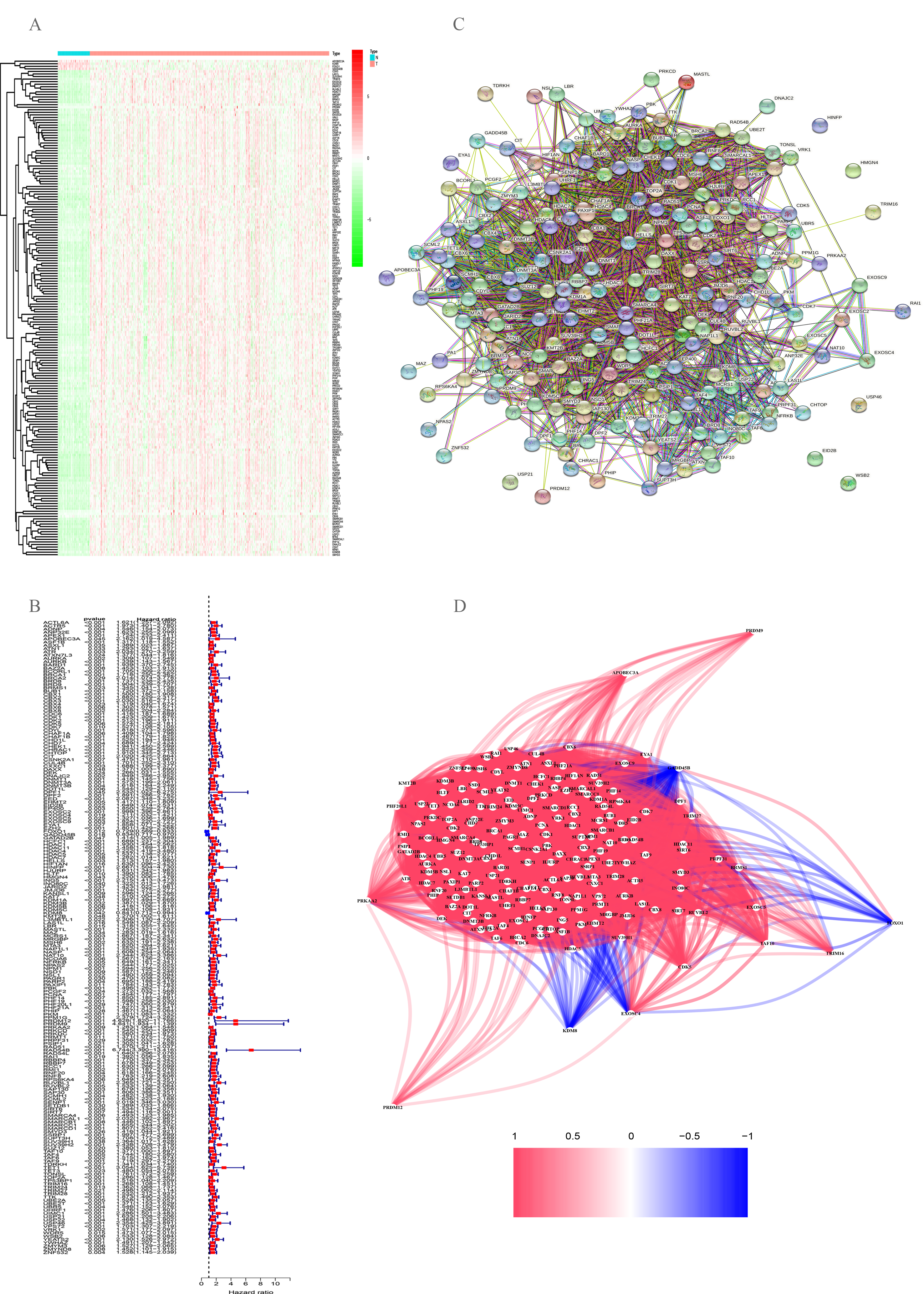

Supplement: Supplementary file 3 [file Image1.TIF]
